# Clinical impact of 16S rRNA RC-PCR NGS on infectious disease management

**DOI:** 10.1128/spectrum.00023-26

**Published:** 2026-07-13

**Authors:** Merlijn H. I. van Haren, Lieke ten Have, Pieter D. Koopman, Jochem B. Buil, Ianthe Maat, Janette C. Rahamat-Langendoen, Liesbeth Martens, Simone J. C. F. M. Moorlag, Bart van den Bosch, Ellen Koenraad, Heiman F. L. Wertheim, Willem J. G. Melchers, Suzan D. Pas

**Affiliations:** 1 Department of Medical Microbiology, Radboudumc Community for Infectious Diseases, Radboudumc, Nijmegen, the Netherlands; 2 Clinical Laboratory, Catharina Hospital 3168, Eindhoven, the Netherlands; 3 Clinical Laboratory, Máxima Medical Center, Veldhoven, the Netherlands; UCI Health, Orange, California, USA

**Keywords:** 16S rRNA, molecular diagnostics, NGS, clinical impact, metagenomics

## Abstract

16S rRNA metagenomics provides a culture-independent method for diagnosing infections with fastidious or uncultivable organisms, guiding targeted therapy, and detecting polymicrobial communities. This study utilizes reverse complement (RC)-PCR next-generation sequencing (NGS) to accurately identify bacterial pathogens from clinical specimens and assess its impact on clinical decision-making, setting it apart from conventional 16S sequencing approaches. A retrospective analysis of an ISO 15189 accredited 16S RC-PCR NGS diagnostic workflow targeting the V1–6 and V9 regions of the 16S rRNA gene was conducted over a 2-year period, including 390 clinical specimens from 316 patients. 16S RC-PCR NGS results were discussed in a multidisciplinary consultation and subsequently reported to the clinic. In total, 1,283 RC-PCR results were analyzed, of which 517 were from clinical specimens, 284 were negative controls, 66 were positive controls, and 416 were from wet lab and bioinformatic pipeline validation. 16S RC-PCR NGS assay detected bacterial taxa in 179/390 (45.9%) of clinical specimens, while 201/390 (51.5%) were negative, and 10/390 (2.6%) yielded uninterpretable results. The specimen types pus, pleural fluid, and heart valves exhibited the highest positivity rate (68% to 70%). Overall, 16S RC-PCR NGS influenced diagnostic decision making in 145/282 (51.4%) clinical cases and guided therapeutic management in 77/282 (27.3%) cases. Results providing definite evidence for either the presence or absence of bacterial infection were considered clinically valuable. Integration of 16S RC-PCR NGS pathogen detection with multidisciplinary consultation markedly improved clinical management, directly impacting diagnosis and treatment of complex clinical cases in a tertiary care setting. The effect was most pronounced in brain abscess patients, where RC-PCR results guided treatment decisions in 9/13 (69.2%) of cases.

**IMPORTANCE:**

Timely and accurate diagnosis is essential for managing serious infections, yet clinicians often face situations where routine laboratory tests do not provide clear answers. This study demonstrates that next-generation sequencing (NGS) of the bacterial 16S rRNA gene can decisively resolve these uncertainties. By revealing whether bacteria are present in clinical specimens, this approach influenced clinical reasoning and supported treatment decisions across a variety of challenging cases. 16S reverse-complement PCR was especially powerful for brain abscesses and infections where the causative microorganism was unclear, providing clarity that directly improved patient care. These findings show that integrating advanced sequencing with expert clinical interpretation can enhance the management of complex infections and support more confident, evidence-based therapy.

## INTRODUCTION

Infections caused by bacterial pathogens are a major global health concern, contributing to an estimated 7.7 million deaths annually (1). Accurate and timely identification of these pathogens is critical for effective patient treatment. Traditionally, culture-based methods have been the gold standard for bacterial identification. These methods are robust, relatively inexpensive, and allow for direct antibiotic susceptibility testing, which is crucial for guiding treatment decisions (2). A challenge in bacterial culture is the limited ability to successfully grow a range of clinically relevant bacterial species under standard laboratory conditions. Additionally, specimens taken after administration of antibiotics often exhibit impaired growth. These factors can lead to false-negative results or biased identification in cases of mixed infections (3, 4).

To overcome these challenges, molecular techniques like PCR and subsequent next-generation sequencing (NGS) are increasingly used to identify bacterial pathogens directly from clinical samples. While these methods offer advantages, prior knowledge of the suspected pathogen is required. Sequencing the 16S rRNA gene offers a powerful alternative for broad-range bacterial identification. This gene is highly conserved across 96% of bacterial genera, yet contains nine species-specific hypervariable regions (V1 to V9) flanked by conserved sequences, ideal for primer design (5). 16S rRNA gene Sanger sequencing has been widely adopted by microbiology laboratories, providing higher resolution for bacterial identification compared to other broad identification methods like MALDI-TOF (6). However, Sanger sequencing struggles with polymicrobial samples, is labor-intensive, and sensitivity is limited when applied directly to clinical samples (7).

Recent advances led to the development of 16S reverse complement PCR (16S RC-PCR) targeting the V1–V6 and V9 regions, followed by NGS on short-read (Illumina) platforms (8). During the PCR, barcode-containing amplicons are formed, substantially reducing the library preparation. With increased sensitivity compared to both Sanger sequencing and traditional culture, this technology significantly impacts pathogen identification, particularly in complex clinical scenarios (8).

This study evaluates the technical and clinical performance of 16S RC-PCR NGS over a 2-year period (April 2023 to April 2025). Clinical specimens (e.g., bone tissue, cerebrospinal fluid [CSF], pus) were processed using the 16S RC-PCR NGS workflow. Sequencing results and clinical context were reviewed in a multidisciplinary consultation (MDC) before reporting. During this period, workflow improvements, including database expansion and incorporation of internal and positive controls, and the introduction of cut-off values to improve inhibition and QC monitoring, were implemented. The MDCs ensured consistent interpretation throughout changes. Here, we present the diagnostic performance of the 16S RC-PCR NGS assay, the impact of workflow modifications, and the clinical utility of this method.

## MATERIALS AND METHODS

### Data collection and inclusion

Data were retrospectively collected from the Laboratory Information Management System (LIMS) and supplemented with electronic patient records when required. All 16S RC-PCR NGS results from clinical specimens tested between 1 April 2023 and 1 April 2025 were included for technical analysis (517 tests on 390 specimens; Fig. S1a). For clinical analysis, 3 tests not requested by the clinic, 27 external hospital cases, and 4 cases with insufficient data were excluded. Additionally, 132 specimens from 58 patients were grouped, resulting in 282 included cases (Fig. S1b). Collected variables included 16S RC-PCR NGS technical data, specimen characteristics, microbiological test results (all microbiological tests as performed during standard of care, including bacterial and fungal cultures, molecular testing, antigen tests, and serology), patient characteristics (age, gender, working diagnosis), and clinical impact (diagnostic conclusions and treatment changes based on 16S RC-PCR NGS assay). Diagnostic and treatment impact were categorized as positive, neutral, or no impact based on specific categories.

### Impact on diagnoses and treatment

Diagnostic impact was categorized as positive for the identification of a microorganism not found before (A), identification of a microorganism found before in a different type of material with uncertain involvement in infection (B), identification of a microorganism found before, which contributed to ruling out additional pathogens (C), and negative 16S RC-PCR NGS, which contributed to ruling out bacterial infection (D). 16S RC-PCR NGS was considered neutral when a microorganism was identified, which was already found in the same material by another method (E), and when 16S RC-PCR NGS was negative, while a pathogen was identified with different diagnostics (F). 16S RC-PCR NGS assay had no impact when the diagnosis remained infection despite negative 16S RC-PCR, even if no other pathogen was found (G), and for all other cases (H). See Table S1 for a full overview.

Treatment impact was categorized as positive when it led to continuation of empirical treatment (A), optimization of antibiotic treatment (B), or stopping of antibiotic treatment (C) based on the 16S RC-PCR NGS result. Treatment impact was neutral when treatment decisions had already been made before results of the 16S RC-PCR NGS assay were available (D). Other cases were considered to have no treatment impact (E). See Table S2 for a full overview.

### 16S rRNA RC-PCR protocol

The ISO15189:2021 accredited 16S RC-PCR short-read amplicon-metagenomic workflow, including DNA extraction, library preparation, and NGS, was performed as previously described, with minor additions (Appendix I) (8). From April 2024 onward, 20 µL 10× diluted Spike-in Control II (Low Microbial Load, D6321, ZymoBIOMICS) in PBS was added to 200 µL clinical specimen and negative extraction control (PBS) before DNA extraction (“internal control”). To detect kitome background signals, a negative PCR control was included in each run. At least once a month, a positive control of 1 µL 10× diluted Microbial Community DNA Standard II (D6311, ZymoBIOMICS) in DPEC water was included. The internal control, with a theoretical final load of *Truepera radiovictrix*, *Imtechella halotolerans*, and *Allobacillus halotolerans* at 2.0 × 10^4^, 3.0 × 10^3^, and 7.0 × 10^2^ 16S copies per sample (assuming 100% DNA extraction efficiency), respectively, served as an inhibition criterion; detection of *Truepera radiovictrix* and *Imtechella halotolerans* above 0.01 abundance (calculated as assigned paired-end reads/total assigned paired-end reads) was required to report negative results. For the positive control, detection of *Pseudomonas aeruginosa* (theoretical abundance 0.028) indicated a successful run.

An in-house bioinformatic pipeline (https://github.com/Bioinf-MMB-Rumc/RC-PCR_16S) was used to process sequencing reads using the 16S rRNA SILVA database (SSURef NR99 release 138.1, downloaded 17-01-2023) (9) as the primary reference for taxonomic assignment. From June 2023 onward, the NCBI RefSeq 16S rRNA (10) (bioproject 33175 and 33317, downloaded 05-06-2023) (10) was additionally used for secondary evaluation of ambiguous classifications. The pipeline generated a sample report containing general QC metrics, reads per amplicon, and assigned taxa per database (Fig. S2). The SILVA database was used as the primary database for taxonomic identification; RefSeq was used for confirmation. Subsequently, results were discussed in an MDC, including at least a clinical microbiologist, a bioinformatician, a medical molecular microbiologist, and a laboratory technician, before reporting clinically relevant bacterial species (Fig. S3) (8). The bioinformatic pipeline reported a taxon if all predefined thresholds were met: relative abundance exceeded 0.01, assigned reads exceeded 100, amplicon number exceeded 3, and breadth of coverage of the 16S gene exceeded 30%. Species-level reporting was performed when taxonomic assignment was considered robust based on the concordance of read assignments and known discriminatory capacity. In cases where reads mapped to multiple species within the same genus, or when species-level discrimination was considered unreliable, results were reported at the genus level only. The final reporting level was additionally reviewed during MDC in the context of clinical plausibility and known limitations of 16S-based taxonomic resolution.

Following implementation of the internal control, taxon detection required abundance exceeding that of *Allobacillus halotolerans* (112 16S copies assuming 100% DNA extraction efficiency) and absence from the negative PCR and negative isolation control within the same 16S RC-PCR NGS run. Results were classified as technically negative if no taxa were detected or when all detected taxa were also present in the negative control. Technical negatives with *Truepera radiovictrix* and *Imtechella halotolerans* abundance >0.01 were considered valid; otherwise, results were deemed not interpretable (NTI) and repeated using fivefold dilution.

Negative controls were interpreted in a taxon-specific manner. Detection of a taxon in the negative control (e.g., *Escherichia coli*) prompted additional scrutiny of clinical specimens containing the same taxa (e.g., *E. coli*) within the same 16S RC-PCR NGS run. In such instances, relative abundance, total read count, breadth of coverage, and clinical context were evaluated collectively. For example, low-abundance detections comparable to negative control levels were interpreted as negative, whereas 10× higher abundance signals in clinical specimens with all V-regions covered were considered as true positives. Analytical positives were considered clinically irrelevant when sample (site) contamination was suspected or when non-pathogenic taxa were identified and were reported with interpretation comments indicating uncertain clinical relevance. All cases were evaluated during the MDC to reach consensus.

## RESULTS

**Fig 1:**
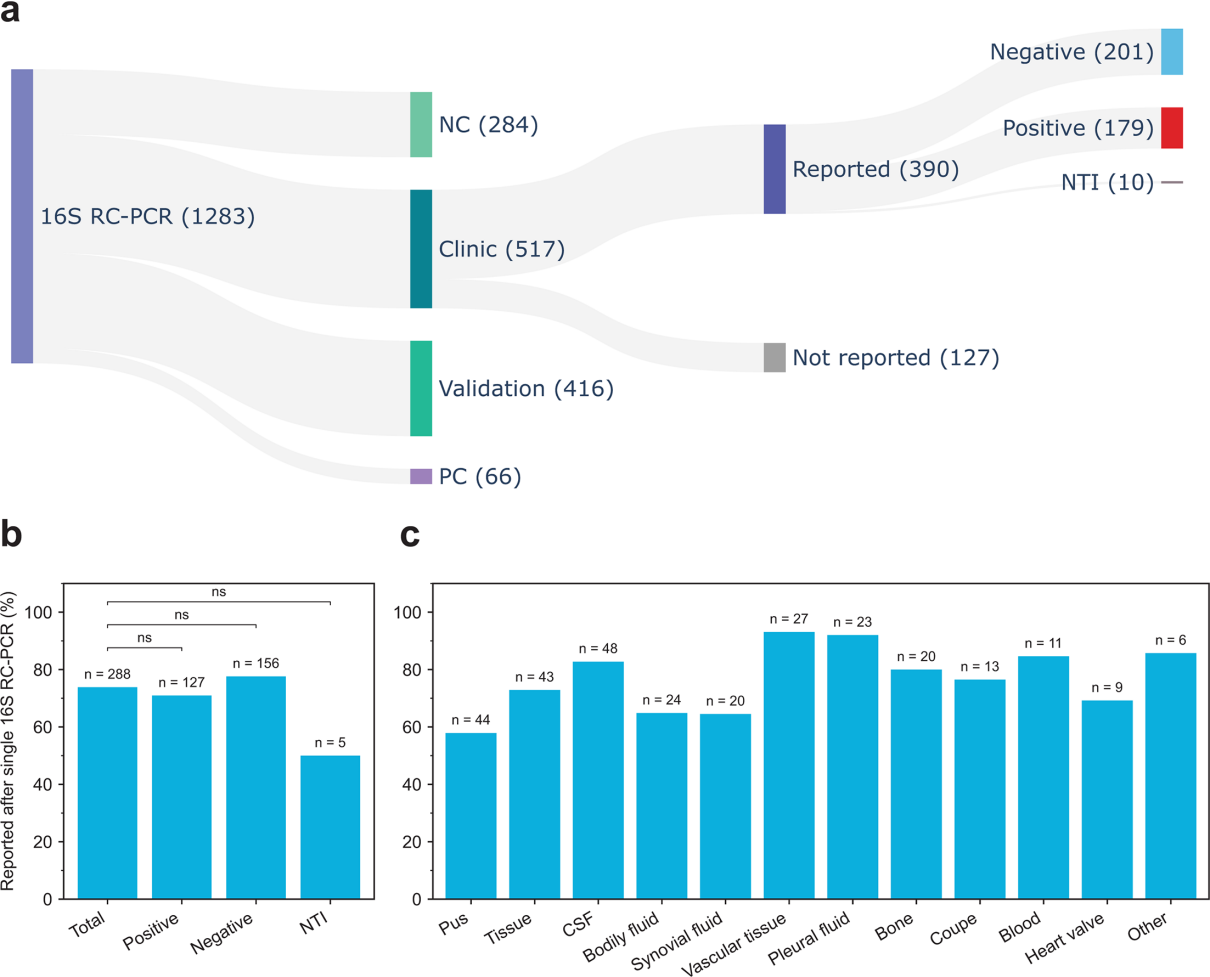
Overview of 16S RC-PCR application over 2 years. (**a**) Of 517 16S RC-PCR NGS assays on clinical specimens, 390 yielded a reported result. (**b**) A total of 102 specimens required repeat testing before a result was obtained. (**c**) Repeat testing by specimen type; *n* indicates the number of tests not requiring repetition. Significance was assessed using Fisher’s exact test. Pus, tissue, and bodily fluid include multiple specimen types; see Fig. S4 for a full overview.

Over 2 years, the 16S RC-PCR NGS assay was performed on 390 clinical specimens of 316 patients, corresponding to 517 tests. Additionally, 66 positive controls, 284 negative controls, and 416 16S RC-PCR NGS assays dedicated to wet-laboratory and bioinformatic pipeline validation were run (Fig. 1a). Pipeline validation samples included various cultured microorganisms, as well as clinical specimens, such as CSF, synovial fluid, pus, heart valves, bone, and other tissues (8).

### Clinical specimens analyzed

A total of 517 16S RC-PCR NGS assays were performed to obtain reportable outcomes for 390 clinical specimens, as NTI results required repetition. In 288/390 (73.8%) clinical specimens, a reportable result was obtained after a single test, whereas 102/390 (26.2%) underwent two or more 16S RC-PCR NGS analyses. Retrospective analysis showed that only 44/390 (11.3%) specimens would ultimately require repeat testing. During the study period, laboratory technicians started prospectively diluting samples according to material specifications, such as viscosity, to reduce the likelihood of repetitive testing and improve turnaround times. Clinically relevant bacterial taxa were detected in 45.9% of all reported clinical specimens, and 2.6% remained NTI despite repeated analysis (Fig. 1a). Notably, there was no correlation between 16S RC-PCR NGS outcome and the likelihood of requiring repeated analysis (Fig. 1b).

To identify factors contributing to repeat testing, specimen type was examined. Pus, bodily fluid, and synovial fluid showed repeat rates exceeding 30% (Fig. 1c). Subsequent fivefold dilution effectively mitigated PCR inhibition and yielded interpretable results, except for 10 specimens deemed NTI. By contrast, no more than 10% of pleural fluid and blood vessel wall specimens required repeat testing.

### Background detection and controls

**Fig 2:**
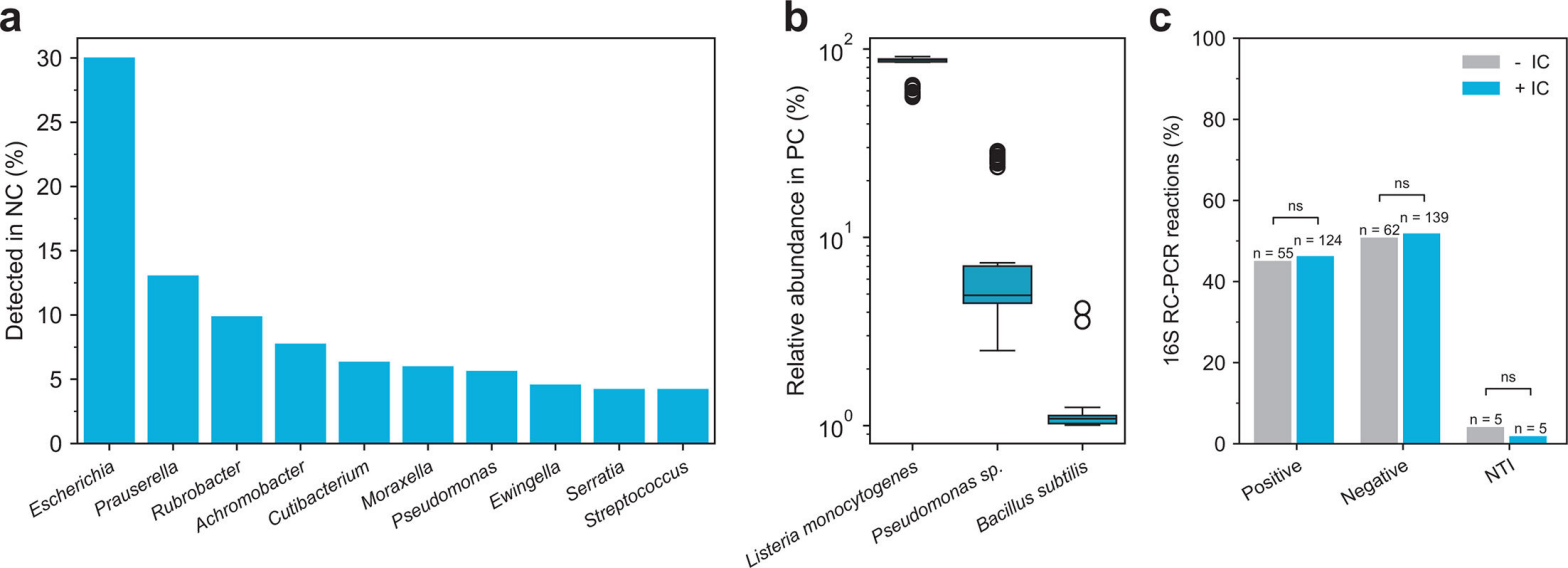
Background detection and controls in 16S RC-PCR NGS assays. (**a**) Ten most frequently detected bacterial taxa in 284 NCs. (**b**) Taxa detected in PCs. Relative abundance calculated as (mapped reads per taxon/total QC-passed reads) × 100. (**c**) Comparison of results before (− IC) and after (+ IC) IC introduction. Significance assessed using Fisher’s exact test.

Across 284 negative controls (NCs), recurrent bacterial taxa were detected, most frequently *Escherichia coli* (30.0%) and *Prauserella isguenensis* (13.1%) (Fig. 2a). These taxa often persisted over consecutive months before disappearing, suggesting (reagent) batch-related contamination (Fig. S5). Sixty-six positive controls (PCs) confirmed assay sensitivity, with all assays meeting detection criteria and demonstrating reliable detection of log-distributed DNA input (Fig. 2b). From April 2024, inclusion of an internal control (IC) standardized reporting thresholds in clinical specimens, representing an assay modification which may affect comparability with earlier analyses. However, Fig. 2c shows no significant changes in 16S RC-PCR NGS outcomes after IC addition. Sensitivity remained unaffected, as IC DNA added to PCs was not detected, consistent with its low abundance/concentration.

### 16S RC-PCR NGS bacterial taxa detection

**Fig 3:**
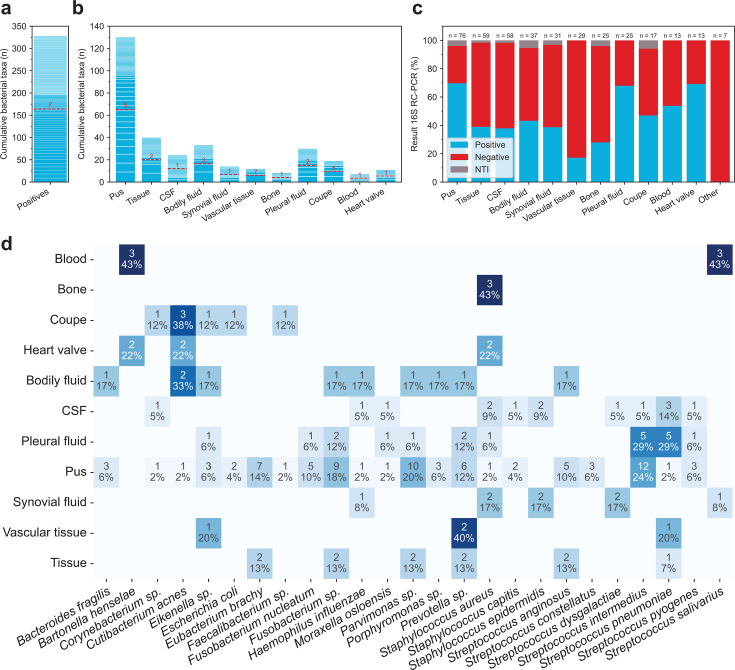
Bacterial taxa detected by 16S RC-PCR NGS assay. (**a**) Total number of taxa identified in positive clinical specimens. (**b**) Number of taxa per specimen type. (**c**) Positivity rate per specimen type; *n* indicates the number of samples tested. Pus, tissue, and bodily fluid include multiple specimen types (Fig. S6). (**d**) Detection frequency and within-sample occurrence (%). Counts and percentages indicate detection frequency and sample occurrence, respectively. Only taxa detected in >3 specimens are shown at the species or genus level.

Among the 179 specimens positive for clinically relevant bacterial pathogens, 47 (26.3%) were polymicrobial, with a weighted median of two bacterial taxa detected per specimen (Fig. 3a). The frequency of samples containing more than one taxon varied by specimen type, with pus and histology coupes showing a weighted median of five taxa (Fig. 3b). Across all clinical specimens, 131 distinct bacterial taxa were identified, of which 91 (69.5%) were resolved to species level and 40 (30.5%) to genus level only. In cases where reads mapped to multiple species within the same genus, when the database matches were limited to genus level, or when species-level discrimination was deemed unreliable, results were reported at genus level only. *Streptococcus intermedius* was the most frequently identified species, present in 18 specimens (4.6%, Table S3).

Detection rates varied across specimen types. Pus, pleural fluid, and heart valves yielded the highest positivity rate, all over 65% (Fig. 3c). Within positive pus and pleural fluid specimens, *Streptococcus intermedius* was found most often, 24% and 29%, respectively (Fig. 3d).

### Clinical cases

Impact of the 16S RC-PCR NGS assay on diagnosis and treatment was assessed per clinical case; multiple specimens addressing the same clinical question were grouped. The 390 clinical specimens thus originated from 316 cases (Fig. S1b). Thirty-four cases were removed: 3 because 16S was not performed by clinical request, 4 because there was insufficient data to determine the clinical impact, and 27 cases that belonged to external hospitals. The remaining 282 cases covered diverse working diagnoses, most commonly meningitis (37 cases, of which 5 were drain-related), infectious aortitis (36, of which 20 prosthetic-related), and pulmonary infections (22 cases). Other notable groups are endocarditis (15 cases, of which 12 prosthetic valve endocarditis) and brain abscesses (13 cases). Gender distribution was skewed toward males (182 vs 100), especially in endocarditis (15 cases, all male) and prosthetic-related aortic infections (18 males, 2 females). The median age was 59 years (range 0–90).

### Impact on diagnosis

**Fig 4:**
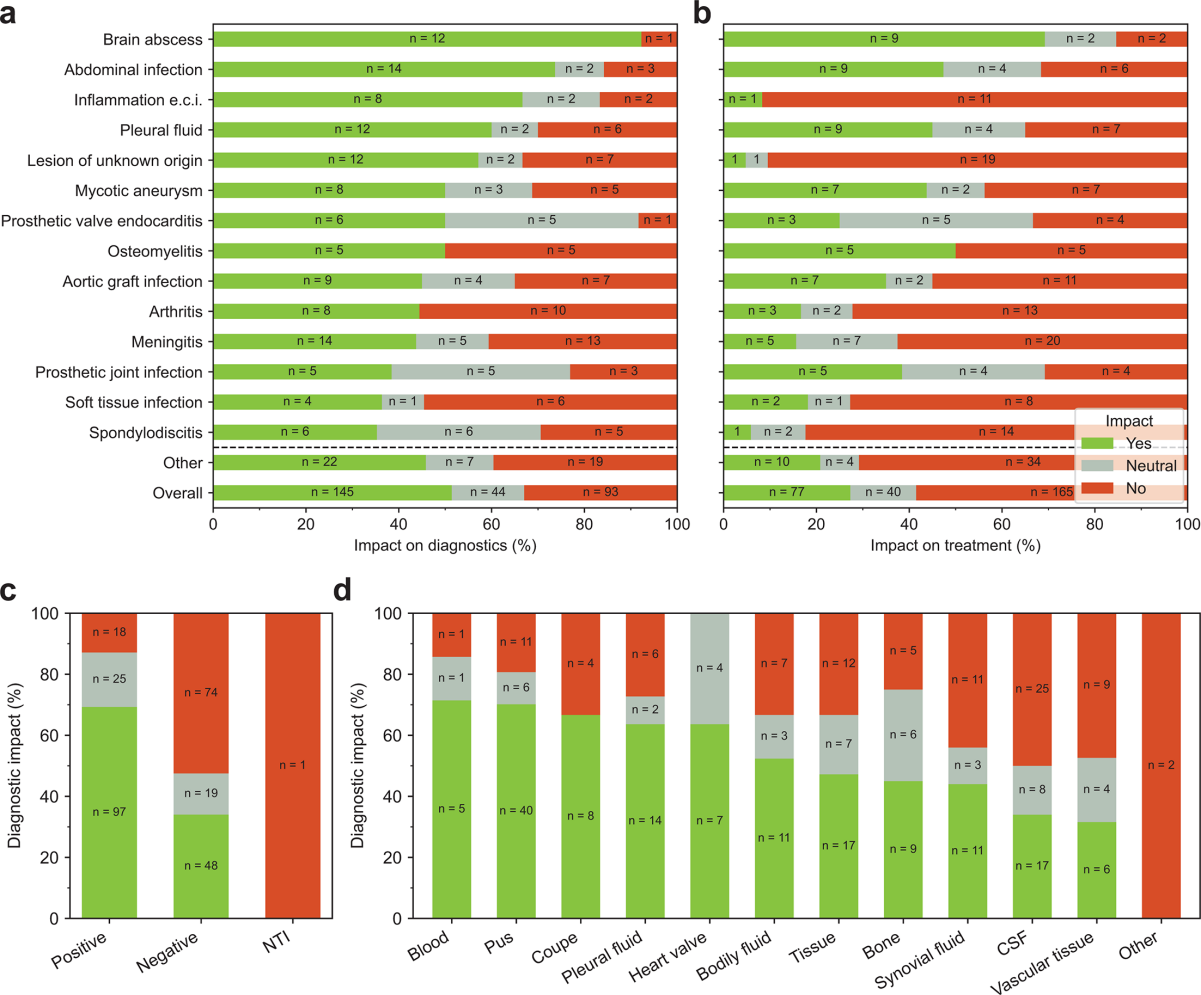
Diagnostic and therapeutic impact of 16S RC-PCR NGS assay. Impact on diagnosis (**a**) and treatment (**b**) shown for all cases (overall, bottom row) and diagnostic groups with >10 cases (groups with fewer cases are combined in “Other,” the full data set is available in Fig. S7 and S8). Diagnostic impact stratified by 16S RC-PCR NGS result (**c**) and specimen type (**d**).

16S RC-PCR NGS impacted clinical diagnosis in 145/282 cases (51.4%) (Fig. 4a), mainly by identifying pathogens missed by conventional methods (category A), and by contributing to ruling out bacterial infection (category D) (both 61 cases, 21.6%). In 15 cases (5.3%), 16S RC-PCR NGS confirmed microorganisms previously detected in other materials, and in 8 cases (2.8%), it ruled out additional pathogens. Impact varied by diagnostic group (>10 patients), from 35.3% in spondylodiscitis to 92.3% for brain abscesses (Fig. 4a). All diagnostic groups except for sepsis (two patients) had at least one useful result.

16S RC-PCR NGS had a neutral impact on diagnosis in 44 cases (negative 16S RC-PCR NGS, pathogen was found by a different method [23 cases, 8.2%] or confirmation of a cultured microorganism [21 cases, 7.4%]). Furthermore, 93 cases showed no diagnostic impact, notably 40 cases (14.2%) where bacterial infection remained the working diagnosis despite negative 16S RC-PCR. These 40 cases contained diverse working diagnoses and sample types, without clear similarities.

### Impact on treatment

16S RC-PCR NGS positively impacted treatment in 77/282 cases (27.3%) (Fig. 4b): treatment was optimized in 36 cases (12.8%), empirical treatment was correct and continued in 26 cases (9.2%), and antibiotic treatment was discontinued in 15 cases (5.3%) after a negative result. In 40 cases (14.2%), 16S RC-PCR NGS had a neutral impact on therapeutic management, defined as a potentially useful result, but treatment decisions had already been made before results were available. 16S RC-PCR NGS had no impact on treatment in the remaining 165 cases (58.5%). Therapeutic impact varied per diagnostic category. In diagnostic categories with >10 patients, brain abscesses showed the highest impact on treatment (9/13, 69.2%), mainly confirming correct empirical treatment or allowing treatment optimization. The lowest treatment impact was found in lesions of unknown origin (1/21, 4.8%) and spondylodiscitis (1/16, 6.3%). The two cases with impact involved cessation of antibiotic treatment after a negative 16S RC-PCR.

Diagnostic groups where 16S RC-PCR NGS had no positive impact on treatment were pericarditis (seven patients), drain-related meningitis (five patients), neurological deficiency e.c.i. (three patients), pneumonia (two patients), and sepsis (two patients) (Fig. S7 and S8). As expected, positive 16S RC-PCR NGS assays impacted diagnosis or treatment more often than negative results (72.5% vs 33.3%, Fig. 4c). Blood, pus, coupes, pleural fluid, and heart valves had the highest diagnostic impact (Fig. 4d).

## DISCUSSION

Our study establishes that 16S RC-PCR NGS exerts a substantial influence on both diagnostic and therapeutic decision-making, impacting 51.4% and 27.3% of cases of 282 patients over two years. Crucially, the 16S RC-PCR NGS assay was deployed as a tertiary diagnostic modality, primarily when standard of care testing yielded inconclusive results, highlighting its role as a problem-solving tool, rather than a first-line assay. Interpretation within an MDC allowed integration of sequencing data with clinical context, optimizing both accuracy and relevance.

While the SILVA database served as the primary reference for 16S taxonomic assignment due to its redundant nature, its moderate curation and inclusion of environmental or difficult-to-culture taxa may compromise clinical interpretability (11). To improve confidence in ambiguous classifications, the NCBI RefSeq 16S rRNA database was introduced in June 2023 as a secondary, highly curated, and non-redundant reference database (10). In cases where SILVA- and RefSeq-based assignments remained discordant or uncertain, additional reflex analysis using Kraken was performed to further resolve ambiguous taxa (12). Despite broad bacterial detection capabilities, species-level identification using 16S sequencing remains limited for certain taxa with highly conserved ribosomal regions. Therefore, some organisms were reported at the genus level when species-level discrimination was considered unreliable or clinically implausible. Looking forward, long-read sequencing methods like Oxford Nanopore Technology (ONT) offer the potential to substantially improve species-level classification, addressing one of the principal limitations of short-read 16S diagnostics and enabling more precise therapeutic targeting (13).

Adding appropriate concentrations of internal process controls improved inhibition detection and enabled clearer discrimination between genuine infection and process contamination (e.g., *Escherichia coli* and *Achromobacter insuavis* in negative controls) after April 2024. This distinction was particularly relevant in specimens susceptible to environmental or collection-derived contaminants, including fecal-associated taxa detected in neonatal lumbar punctures.

During the study period, prospective dilution of viscous specimens was also introduced to reduce NTI outcomes. Consequently, part of the observed repeat-testing frequency reflected pre-emptive workflow optimization rather than true assay failure, which should be considered when interpreting repeat-analysis rates. Furthermore, the introduction of the IC represents a modification that may affect comparability with data generated prior to its implementation, particularly with respect to interpretation by MDC. Analysis showed that the IC addition did not influence taxa detected in the positive control, suggesting that assay sensitivity was not compromised. Nevertheless, subtle effects on reporting thresholds or interpretation cannot be excluded. Overall, this refinement supports a more consistent interpretation of results and strengthens confidence in the 16S RC-PCR NGS assay as a diagnostic tool. These findings also highlight the need for standardized guidance regarding internal controls, contamination thresholds, database selection, and interpretation frameworks for clinical microbiological NGS workflows, particularly as such assays become more widely implemented in routine diagnostics.

Interpretation of 16S RC-PCR NGS results relied on integration of analytical thresholds, negative-control comparison, and clinical context rather than fixed quantitative cutoffs alone. Unlike untargeted metagenomic approaches, RC-PCR includes targeted amplification steps that may introduce primer- and amplicon-dependent biases, limiting direct quantitative interpretation of read abundance. Therefore, clinically relevant taxa were identified using a standardized multi-parameter framework reviewed within MDC. The limit of detection of the 16S RC-PCR NGS assay was established between 47.2 and 4.6 cells for *E. coli* based on an internationally available control, which will differ for other species depending on DNA extraction efficiency and primer specificity (8). The resolution afforded by 16S RC-PCR NGS was particularly evident in polymicrobial specimens (47/179, 26.3%), detecting up to nine taxa per sample, a clear advantage over conventional Sanger sequencing. Purulent specimens exhibited a median of five taxa, with *Streptococcus intermedius* predominating, consistent with its established role in abscess formation (14). The ability to resolve polymicrobial infections not only facilitated pathogen detection but also contributed to ruling out additional pathogens in eight cases, each contributing positively or neutrally to clinical management.

When contextualized within the literature, our findings underscore the substantial added value of the 16S RC-PCR NGS assay. Prior studies using Sanger sequencing report diagnostic and therapeutic impacts ranging from <10% to 45.9%, whereas NGS-based studies targeting V1–V3 regions only demonstrate markedly higher positivity, albeit without uniform translation into therapeutic decision-making (7, 15–18). The 27.3% treatment impact observed here cannot be attributed solely to NGS methodology, but reflects the combined effect of specimen selection, interpretive rigor, and MDC (19). Indeed, clinical impact was highly dependent on the diagnostic context: in lesions of unknown origin, 16S RC-PCR NGS predominantly contributed to ruling out bacterial infection (57.1% diagnostic impact) with minimal therapeutic change, whereas in brain abscesses, it confirmed empirical therapy (38.5%) or guided treatment (30.8%) in the majority of cases. These observations highlight the interplay between pathogen biology, patient factors, and clinical decision-making in determining the utility of advanced molecular diagnostics.

Within this hospital, 16S RC-PCR NGS assay is used as a last-line diagnostic assay to resolve complex cases. In this setting, clarifying the involvement of a microorganism in a specific location or confirming negative results can be seen as a positive impact of the test in certain cases, even if this has no therapeutic consequence. When only considering cases where 16S RC-PCR NGS assay allowed bacterial identification when standard of care testing did not (61/282 cases, 21.6%) for diagnostic utility and antibiotic optimization (51/282 cases, 18.1%) for therapeutic utility, the impact numbers are lower. This reiterates that the impact of 16S RC-PCR NGS diagnostic testing is dependent on the indication for testing and the timing of the assay, aside from merely test characteristics. Due to its retrospective real-world study design in a highly selected tertiary-care cohort, no formal comparator or control group was included, precluding direct quantification of incremental diagnostic performance.

No definite conclusions could be drawn on the clinical impact of 16S RC-PCR NGS assay for different diagnostic groups, as each group with more than two cases had at least one case with a positive impact on diagnosis, and most groups had at least one case with a positive impact on treatment. Indeed, the contribution of 16S RC-PCR NGS to clinical management is case-specific, which is why we recommend implementing the 16S RC-PCR NGS assay for specific cases where conventional diagnostics yield inconclusive results, rather than implementing it as a first-line assay.

16S RC-PCR NGS remains resource-intensive, and sequencing runs required a minimum batch size of eight samples, including two controls, in our setting. Bench costs were estimated at approximately 300 euros per clinical specimen under routine low-volume conditions. Cost efficiency improves substantially with larger batch sizes and could be further improved by combining multiple assays on a single Illumina flowcell, considering the difficulties with distinct barcoding and different downstream bioinformatic pipelines. Implementation may be most feasible in tertiary or centralized laboratories with sufficient sample throughput and multidisciplinary expertise for interpretation. Long-read sequencing, e.g., ONT, may further improve scalability and accessibility by reducing costs and enabling more flexible flowcell utilization. Turnaround time remains a key limitation: results were available no earlier than 3 days and could require up to 10 days for the regular workflow. Repeat testing was required in 16.0% of cases, particularly for pus, bodily fluid, and synovial fluid (>30%), substantially impairing real-time clinical applicability. Pre-dilution of these specimen types improved diagnostic efficiency and may reduce the need for retesting. Notably, in 40 cases (14.2%), therapeutic decisions had been made before 16S RC-PCR NGS results were available. In this group, improved turnaround time could significantly improve impact, underscoring the importance of workflow optimization and rapid reporting.

In conclusion, 16S RC-PCR NGS assay significantly enhances diagnostic precision and informs therapeutic decisions in complex, culture-negative, or polymicrobial infections. Its greatest value lies in resolving ambiguous cases, detecting multiple pathogens within a single specimen, and guiding targeted treatment, particularly for difficult-to-culture organisms. Maximizing clinical impact requires careful case selection, rapid turnaround, and multidisciplinary interpretation, while integration of long-read sequencing and curated reference databases promises further gains in taxonomic resolution and actionable insights. Collectively, these findings highlight the potential of 16S RC-PCR NGS as a valuable tool in modern clinical microbiology, supporting the integration of molecular detection with informed patient management.

## Data Availability

Data used for analyses and figures are available upon reasonable request.

## SUPPLEMENTAL MATERIAL

File S1 spectrum.00023-26-s0001.xlsx Diagnostic data.

Supplemental material spectrum.00023-26-s0002.docx Fig. S1 to S8, Tables S1 to S3, and Appendix I.

ASM does not own the copyrights to Supplemental Material that may be linked to, or accessed through, an article. The authors have granted ASM a non-exclusive, world-wide license to publish the Supplemental Material files. Please contact the corresponding author directly for reuse.
