# Supplementary material for: Clinical impact of 16S rRNA RC-PCR NGS on infectious disease management
Source: Microbiol Spectr. 2026 Jul 13;14(8):e00023-26. doi: 10.1128/spectrum.00023-26 (PMC13436334; doi:10.1128/spectrum.00023-26)
Supplement: Supplementary file 1 [file spectrum.00023-26-s0002.docx]

**Supplementary information**

**Clinical impact of 16S rRNA RC-PCR NGS on infectious disease management**

Merlijn H. I. van Haren^1†#^, Lieke ten Have^1†^, Pieter Koopman^1^, Jochem B. Buil^1^, Ianthe Maat^1^, Janette C. Rahamat-Langendoen^1^, Liesbeth Martens^1,2,3^, Simone J. C. F. M. Moorlag^1^, Bart van den Bosch^1^, Ellen Koenraad^1^, Heiman F. L. Wertheim^1^, Willem J. G. Melchers^1^, Suzan D. Pas^1^

^1^Department of Medical Microbiology, Radboudumc Community for Infectious Diseases, Radboudumc, Nijmegen, The Netherlands

^2^Clinical Laboratory, Catharina Hospital, Eindhoven, The Netherlands

^3^Clinical Laboratory, Máxima Medical Center, Veldhoven, The Netherlands

**^#^Correspondence:** Dr. Merlijn H. I. van Haren

^†^Both authors contributed equally

Contains **Supplementary Figures 1-8** and **Supplementary Tables 1-3** and **Appendix I**

**Supplementary Figure 1** Flowchart for 16S RC-PCR NGS assay data inclusion in technical (**a**) and clinical (**b**) analysis. Blue indicates steps used to filter the total dataset, red indicates unreported results, and purple shows the grouped results, specifically for the clinical samples that originate from a single patient.

**Supplementary Figure 2** 16S RC-PCR NGS assay report. (**a**) Overview sheet with quality control parameters and Classification of hits with an abundance over 0.01 to the SILVA and REFSEQ database. *Streptococcus intermedius* was detected in this specimen. (**b**) Taxa detected with an abundance < 0.01. *Truepera radiovictrix*, *Imtechella halotolerans*, *Allobacillus halotolerans* originating from the internal control can be seen. Template: taxonomy of hit in database, Score: Classification score by KMA, Template_coverage: breadth of coverage of 16S gene, Depth: total length of assigned reads divided by 16S length, Q_value: corrected p-value of classification by KMA, FragmentCount: number of paired-end reads assigned to hit, Abundance: relative proportion of assigned paired-end reads to hit (FragmentCount) divided by the total of assigned paired-end reads in sample, V1-V2, ... V9: number of reads assigned to each V-region, Amp_frags: total read assigned to V-regions, Amp_count: number of V-regions with >10 reads assigned, Amp_abundance: relative proportion of reads assigned to hit compared to total reads assigned to hits (together 1.00), Expected: value of expected number of reads when randomly assigned across entire database.

**Supplementary Figure 3** Multidisciplinary consultation (MDC) workflow. 16S RC-PCR NGS reports are inspected during the MDC, after which for each sample a technical and clinical consensus is reached.

**Supplementary Figure 4** Complete overview of repetition of clinical specimens. Pus (pus - other, pus – brain, pus – abscess, pus – abdomen and cyst fluid), bodily fluid (bodily fluid – other, pericardial fluid, ascites fluid and BAL (Bronchoalveolar lavage)) and tissue (tissue – other, skin tissue, joint tissue, lymph node and hematoma) consist of multiple specimens in the main text (**Figure 1c**).

**Supplementary Figure 5** Bacterial taxa found in the 16S RC-PCR NGS assay negative controls over a two-year period. *Escherichia* was consistently found during this period.

**Supplementary Figure 6** Positivity rate of 16S RC-PCR NGS assay per specimen type. *n* indicates number of samples tested per specimen type. Pus (pus - other, pus – brain, pus – abscess, pus – abdomen and cyst fluid), bodily fluid (bodily fluid – other, pericardial fluid, ascites fluid and BAL (Bronchoalveolar lavage)) and tissue (tissue – other, skin tissue, joint tissue, lymph node and hematoma) consist of multiple specimens in the main text (**Figure 3c**).

**Supplementary Figure 7** Impact of the 16S RC-PCR NGS assay on diagnostics (**a**) and treatment (**b**) outcomes. All categories with n < 10 were collapsed in the ‘Other’ category in the main text.

**Supplementary Figure 8** Categories of impact of the 16S RC-PCR NGS assay on diagnostics (**a**) and treatment outcomes (**b**). All categories with n < 10 were collapsed in the ‘Other’ category in the main text.

**Supplementary Table 1** Categories used to define the impact of 16S RC-PCR NGS assay on diagnostic outcome of 282 clinical cases.

| Category | Impact | | Clinical scenarios | Number of cases | % of total |
| --- | --- | --- | --- | --- | --- |
| A | Positive | Identification of a micro-organism that had not been found before. | Conventional microbiological diagnostics are negative; 16S RC-PCR NGS is positive for a pathogen. | 61 | 21.6% |
| B | Positive | Identification of a micro-organism that had been found before in a different material with uncertain involvement in infection. | Microbiological diagnostics are positive, but not in material at the site of infection (e.g. positive blood cultures). 16S RC-PCR NGS does show a pathogen in a sample taken from the site of infection. | 15 | 5.3% |
| C | Positive | Identification of a micro-organism that had been found before in the same material + contributed to ruling out additional pathogens. | Culture is positive for a single micro-organism. 16S RC-PCR NGS is positive for only this micro-organism, after which additional pathogens are ruled out. | 8 | 2.8% |
| D | Positive | Contributed to ruling out (bacterial) infection. | After negative 16S RC-PCR NGS assay, bacterial infection is ruled out. | 61 | 21.6% |
| E | Neutral | Identification of a micro-organism that had been found before in the same material | Culture is positive; 16S RC-PCR NGS is positive for this same micro-organism. | 21 | 7.4% |
| F | Neutral | No impact as the working diagnosis remained infection, detected with different diagnostics. | 16S RC-PCR NGS assay is negative, but conventional microbiological diagnostics show a causative pathogen (including pathogens that 16S RC-PCR NGS assay cannot find, such mycobacteria, fungi and viruses). | 23 | 8.2% |
| G | No | No impact as the working diagnosis remained infection despite negative diagnostics. | All microbiological diagnostics are negative. Even after negative 16S RC-PCR NGS, working diagnosis remained infection. | 40 | 14.2% |
| H | No | No impact of 16S RC-PCR NGS assay | All other scenarios. | 53 | 18.8% |

**Supplementary Table 2** Categories used to define the impact of 16S RC-PCR NGS assay on treatment outcome of 282 clinical cases.

| Category | Impact | | Clinical scenarios | Number of cases | % of total |
| --- | --- | --- | --- | --- | --- |
| A | Positive | Empirical treatment was correct and continued based on 16S RC-PCR NGS assay results. | 16S RC-PCR NGS assay found or confirmed the pathogenic micro-organism(s). The empirical regimen is most optimal for the micro-organism(s) and is continued. | 26 | 9.2% |
| B | Positive | Antibiotic treatment was optimized based on 16S RC-PCR NGS results. | 16S RC-PCR NGS assay found or confirmed the pathogenic micro-organism(s), after which the antibiotic regimen was switched to a more optimal regimen (escalation or de-escalation). | 36 | 12.8% |
| C | Positive | Antibiotic treatment was stopped based on the 16S RC-PCR NGS results. | 16S RC-PCR NGS assay did not show pathogenic micro-organism(s) and antibiotic treatment was stopped. | 15 | 5.3% |
| D | Neutral | Treatment decisions had already been made before results of the 16S RC-PCR NGS assay were available | 16S RC-PCR NGS assay did have a potentially useful result, but no impact on treatment because treatment decisions had been before the 16S RC-PCR NGS assay results were available.  Examples include cases in which antibiotic treatment had already been completed, the culture had become positive, or the patient had died. | 40 | 14.2% |
| E | No | No impact of 16S RC-PCR NGS assay on antibiotic treatment. | All other scenarios. | 165 | 58.5% |

**Supplementary Table 3** All Bacterial taxa found in 390 clinical specimens with 16S RC-PCR NGS assay over a two-year period. 131 different bacterial taxa found in 179 positive clinical specimens (390 total specimens).

| **Taxa** | **n** | **Taxa** | **n** | **Taxa** | **n** | **Taxa** | **n** |
| --- | --- | --- | --- | --- | --- | --- | --- |
| *Streptococcus intermedius* | 18 | *Mycoplasma pneumoniae* | 2 | *Eubacterium rectale* | 1 | *Lactobacillus sp.* | 1 |
| *Parvimonas sp.* | 14 | *Lancefieldella parvula* | 2 | *Helicobacter bilis* | 1 | *Veillonella sp.* | 1 |
| *Fusobacterium sp.* | 14 | *Prevotella intermedia* | 2 | *Xanthomonas phaseoli* | 1 | *Acinetobacter johnsonii* | 1 |
| *Prevotella sp.* | 13 | *Bulleidia extructa* | 2 | *Corynebacterium tuberculostearicum* | 1 | *Raoultella planticola* | 1 |
| *Staphylococcus aureus* | 11 | *Actinomyces sp.* | 2 | *Dermacoccus barathri* | 1 | *Megasphaera sp.* | 1 |
| *Streptococcus pneumoniae* | 11 | *Klebsiella pneumoniae* | 2 | *Shingobium sp.* | 1 | *Neisseria sp.* | 1 |
| *Eubacterium brachy* | 9 | *Prevotella melaninogenica* | 2 | *Streptococcus oralis* | 1 | *Acinetobacter sp.* | 1 |
| *Cutibacterium acnes* | 8 | *Campylobacter curvus* | 2 | *Paludibacteraceae sp.* | 1 | *Actinotignum sp.* | 1 |
| *Streptococcus anginosus* | 8 | *Helicobacter cinaedi* | 2 | *Streptococcus parasanguinis* | 1 | *Aerococcus urinae* | 1 |
| *Fusobacterium nucleatum* | 7 | *Neisseria meningitidis* | 2 | *Streptococcus pseudopneumoniae* | 1 | *Propionimicrobium lymphophilum* | 1 |
| *Eikenella sp.* | 7 | *Cutibacterium sp.* | 2 | *Lactobacillus lactus* | 1 | *Bacillus smithii* | 1 |
| *Haemophilus influenzae* | 6 | *Capnocytophaga canimorsus* | 2 | *Anoxybacillus contaminans* | 1 | *Solobacterium moorei* | 1 |
| *Streptococcus salivarius* | 6 | *Schaalia meyeri* | 2 | *Pseudomonas fluorescens* | 1 | *Chryseobacterium hominis* | 1 |
| *Streptococcus pyogenes* | 6 | *Moraxella sp.* | 2 | *Enterobacter cloacae* | 1 | *Bifidobacterium dentium* | 1 |
| *Bartonella henselae* | 5 | *Enterobacter hormaechei* | 2 | *Virgibacillus carmonensis* | 1 | *Lysinibacillus sp.* | 1 |
| *Escherichia coli* | 5 | *Proteus mirabilis* | 2 | *Rothia mucilaginosa* | 1 | *Dialister sp.* | 1 |
| *Bacteroides fragilis* | 5 | *Aerococcus sanguinicola* | 2 | *Streptococcus mitis* | 1 | *Chlamydia trachomatis* | 1 |
| *Staphylococcus epidermidis* | 4 | *Enterococcus faecium* | 2 | *Faecalibacterium prausnitzii* | 1 | *Atopobium sp.* | 1 |
| *Streptococcus dysgalactiae* | 4 | *Peptostreptococcus sp.* | 2 | *Oribacterium sp.* | 1 | *Peptostreptococcus stomatis* | 1 |
| *Porphyromonas sp.* | 4 | *Streptococcus sanguinis* | 2 | *Corynebacterium kroppenstedtii* | 1 | *Slackia exigua* | 1 |
| *Faecalibacterium sp.* | 3 | *Parvimonas micra* | 2 | *Treponema sp.* | 1 | *Fusobacterium periodonticum* | 1 |
| *Staphylococcus capitis* | 3 | *Campylobacter rectus* | 1 | *Metamycoplasma hominis* | 1 | *Aggregatibacter aphrophilus* | 1 |
| *Streptococcus constellatus* | 3 | *Methylobacterium radiotolerans* | 1 | *Lentimicrobium sp.* | 1 | *Streptococcus gordoni* | 1 |
| *Corynebacterium sp.* | 3 | *Rothia sp.* | 1 | *Streptococcus bovis* | 1 | *Enterococcus faecalis* | 1 |
| *Moraxella osloensis* | 3 | *Clavibacter insidiosus* | 1 | *Capnocytophaga sp.* | 1 | *Bacteroides sp.* | 1 |
| *Treponema lecithinolyticum* | 3 | *Virgibacillus carmonesis* | 1 | *Selenomonas sp.* | 1 | *Ureaplasma parvum* | 1 |
| *Lactococcus lactis* | 3 | *Haemophilus parainfluenzae* | 1 | *Catonella sp.* | 1 |  |  |
| *Aggregatibacter sp.* | 3 | *Pseudomonas syringae* | 1 | *Fusicatenibacter sp.* | 1 |  |  |
| *Gemella sp.* | 3 | *Sphingopyxis sp.* | 1 | *Prevotella baroniae* | 1 |  |  |
| *Dialister pneumosintes* | 3 | *Microbacterium sp.* | 1 | *Bifidobacterium sp.* | 1 |  |  |
| *Fusobacterium necrophorum* | 3 | *Clostridium perfringens* | 1 | *Firmicutes oral* | 1 |  |  |
| *Streptococcus agalactiae* | 3 | *Dysgonomonas mossii* | 1 | *Veillonella parvula* | 1 |  |  |
| *Staphylococcus sp.* | 3 | *Campylobacter gracilis* | 1 | *Enterococcus raffinosus* | 1 |  |  |
| *Campylobacter sp.* | 2 | *Actinomyces israelii* | 1 | *Staphylococcus caprae* | 1 |  |  |
| *Streptococcus sp.* | 2 | *Aggregatibacter actinomycetemcomitans* | 1 | *peptostreptococcus sp.* | 1 |  |  |

**Appendix I Overview of ISO15189-accredited 16S RC-PCR NGS workflow**

The complete 16S RC-PCR NGS workflow, including wet-laboratory processing, sequencing, bioinformatic analysis, and multidisciplinary interpretation, was implemented within an ISO15189:2021-accredited diagnostic framework.

Technical validation of the assay was previously described by Moorlag *et al.*, including assessment of analytical sensitivity, analytical specificity, repeatability, reproducibility, and clinical performance characteristics.[1] Validation experiments included determination of assay sensitivity using defined bacterial DNA input concentrations and evaluation of cross-reactivity and contaminating background taxa using negative controls and mock communities.

Routine quality control included the use of positive controls, negative controls, and internal process controls to monitor amplification efficiency, contamination, and inhibition. Following implementation of the internal control strategy in April 2024, interpretation of results incorporated comparison with negative-control taxa within the same sequencing run to improve discrimination between true-positive findings and background contamination.

Bioinformatic analysis was performed using a standardized and version-controlled pipeline. Database versions were fixed during routine analysis periods to ensure consistency of taxonomic assignment. SILVA SSURef NR99 release 138.1 served as the primary reference database, while RefSeq 16S rRNA databases (bioproject 33175 and 33317) were used for secondary evaluation of ambiguous assignments. Code review procedures, version tracking, and annual audits were used to maintain quality assurance of the bioinformatic workflow.

Clinical reporting was performed within multidisciplinary consultation (MDC), including at least one clinical medical microbiologist, medical molecular microbiologist, bioinformatician and laboratory technician. Per case, sequencing results, specimen type, clinical presentation, microbiological plausibility, and known limitations of 16S-based taxonomic resolution were discussed. 16S RC-PCR NGS results are reported based on the conclusions by the members of the MDC, leading to more standardization and less bias (**Supplementary Figure 3**).

Clinicians received the results of the 16S RC PCR NGS assay as a conclusion text, e.g. ‘16S RC-PCR result: Staphylococcus aureus'. Each result is accompanied by a consultation from the medical microbiologist to further clarify the result if necessary. , MDC discussions consistently involved experienced users of the 16S RC-PCR NGS assay to support interpretation and clinical translation.

The bioinformatic pipeline and associated workflow documentation are publicly available at: <https://github.com/Bioinf-MMB-Rumc/RC-PCR_16S>

[1]	Moorlag SJCFM, Coolen JPM, van den Bosch B, Jin EH-M, Buil JB, Wertheim HFL, et al. Targeting the 16S rRNA Gene by Reverse Complement PCR Next-Generation Sequencing: Specific and Sensitive Detection and Identification of Microbes Directly in Clinical Samples. Microbiol Spectr 2023;11:e0448322. https://doi.org/10.1128/spectrum.04483-22.
